# Collaborative research to respond to the HIV epidemic: a case of Uganda (Makerere University)-Case Western Reserve University Research Collaboration 1988–2021

**DOI:** 10.4314/ahs.v22i2.8S

**Published:** 2022-08

**Authors:** Grace Muzanyi, Justine Nakibuuka, Harriet Mayanja

**Affiliations:** 1 Uganda (Makerere University)-Case Western Reserve University Research Collaboration, Kampala, Uganda; 2 Department of Medicine, School of Medicine, Makerere University College of Health Sciences, Kampala, Uganda; 3 Case Western Reserve University (CWRU), Cleveland, Ohio, USA

**Keywords:** UCWRU, collaboration, training, mentoring

## Abstract

**Background:**

Collaborative research between institutions may not yield results to transform communities. Many research collaborations come to the end of their life time without achieving their originally set goals and with a dearth of community transformation to show for it.

**Objective:**

To delineate and highlight the achievements of the Uganda (Makerere University)-Case Western Reserve University Research Collaboration

**Methods:**

We retrospectively compiled and reviewed the data on research, training and policy impact achievements of the Uganda (Makerere University)-Case Western Reserve University Research Collaboration over a period of 30 years of its existence.

**Results:**

Over the last 35 years, the Uganda (Makerere University)-Case Western Reserve University Research Collaboration trained a total of 104 Ugandans with Masters, PhDs and other varied graduate training programs. More than 70 large tuberculosis/TB+HIV studies were conducted with more than 360 manuscripts published including landmark local and global TB/HIV policy impact publications.

**Conclusion:**

The Uganda (Makerere University)-Case Western Reserve University Research Collaboration has in the past 35 years built the capacity of Ugandan and international students through conducting landmark research, training and mentoring and contributed to TB HIV management policy changes in Uganda.

## Introduction

The Uganda (Makerere University)-Case Western Reserve University Research Collaboration began in 1986 after a presidential invitation to the late Prof. Fred C. Robbins, Case Western Reserve University (CWRU) Professor and Nobel Laureate, to visit Uganda and assist with the then Human Immune Deficiency Virus/Acquired Immune Deficiency Syndrome (HIV/AIDS) epidemic. Prof Robbins and his Uganda colleague's (including Prof Roy Mugerwa, and Dr Edward Mbidde) vision of a multi-disciplinary research collaboration on HIV/AIDS and its complications officially began with grant funding from the United States (US) National Institute of Health (NIH) in 1988 to better understand the new HIV epidemic health issue in Uganda, undertake capacity building in Uganda through research, training and mentoring.

## Methods of the Uganda (Makerere University)-Case Western Reserve University Research Collaboration's approach to training, research and capacity building

In 1986, a memorandum of understanding was initially signed between Makerere University, Case Western Reserve University, Cleveland Ohio and Uganda's Ministry of Health. Over the years, the collaboration has evolved into a multi-project, multi-disciplinary research, training and capacity building organization funded by the US and European grants to collaboration investigators at Makerere University, Joint Clinical Research Center (JCRC) and Case Western Reserve University. The collaboration's focus has expanded into more disciplines including epidemiology, biomedical sciences, clinical trials, nursing, anthropology, bioethics, biomedical engineering, cancer, cardiovascular disease and other related areas. Training has been key for the collaboration with former trainees currently in leadership positions at Uganda ministries, Universities and the private Sector.Makerere University has been the key host Institution for both local and international students who have been trained and mentored through this collaboration. In addition, senior research investigators under Makerere University have significantly contributed to landmark research that has contained the HIV epidemic over the past 35 years

## Results of the collaboration's training, research and capacity building

The collaboration has received ongoing support from the Fogarty International Center for HIV and TB-related training grants, which have been funded continuously since 1989.Over the last 35 years, Uganda (Makerere University)-Case Western Reserve University Research Collaboration has trained a total of 104 Ugandan trainees, graduating with Masters of Public Health, Masters of Epidemiology and Biostatistics, Masters of Science in biomedical science, doctorate of philosophy degrees as well as other varied graduate training programs(table 1). The Doctoral and Masters training were initially in epidemiology and biostatistics with training based mostly in the United States of America (USA), but over time, we have diversified and moved towards a more Uganda based training including the biomedical sciences. In addition, a number of Ugandans have acquired short skills training in research principles, laboratory management, clinical trial management and research administration. As a special category; 11 trainees from Uganda received 2 graduate degrees: Masters and Doctor of philosophy (PhD). Over 99% of the Ugandan graduates are based in Uganda in various academic, administration, health care and policy positions. Some trainees are now faculty in Ugandan academic institutions. In addition, this training which was initially wholly in the USA, moved to a sandwich program, with training conducted concurrently in Uganda and the USA with an aim of having fully Ugandan based training programs in future. The current Makerere Master's and Doctoral Immunology and Microbiology training program at Makerere University are notable in this joint training endeavor.

**Table 1:** Training and capacity building over 35 years of Uganda (Makerere University)-Case Western Reserve University Research Collaboration

Program	Number of trainees	Examples of notable Impact
MSC Epidemiology and statistics	32	Dr. Elioda Tumwesigye is former Ugandan Minister of Health and Minister of Science, Technology and Innovation, Francis Adatu, MBChB, MS is former Head, NLTP
MSC Microbiology	3	Prof Joloba is Dean of Makerere Sch of Biomedical Sciences and consultant to the Ugandan NTRL,Dr Fred bwanga is the current chair of the Makerere College of health Sciences Higher degrees Committee
MSC Immunology	3	Prof Mayanja is former Dean of Makerere School of Medicine
PhD	28	Dr Mupere is the current head department of pediatrics, Makerere College of Health Sciences, Dr Mafigiri is a senior lecturer faculty of social sciences Makerere University, Imaculate Nankya current head of the CFAR lab at the JCRC
MPH	7	Jessica Milman, MPH is the Managing Director for the PATH Center for Vaccine Innovation and Access (CVIA)
MS Health Services Research	7	
MS Bioethics	3	Dr Fred Nakwagala is the current chair of the Mulago Hospital research ethics committee.
MSNursing	1	Maria Louise Walusimbi is a former principal nursing officer, Ministry of Health
**Short courses**		
Research principles	1500	
Laboratory management	500	
Clinical trial management	1000	
**Clinical training**		
MMED	28	Prof Damalie Nakanjako is the current Principal of Makerere College of Health Sciences, Dr Diana Atwine is the current permanent secretary Mistry of health, Dr Andrew Kambugu is the director of the Infectious Disease Institute Mulago.
Clinical fellowship	18	Dr Okuku is an Oncologist at the Uganda Cancer Institute.
Interventional cardiology	2	Drs James Kayima and Emmy Okello are interventional cardiologists at the Uganda heart Institute.
Cancer management	2	Jackson Orem is the current director of the Uganda Cancer Institute.
Laboratory strengthening	10	Sam Ogwang is a former head of the JCRC TB Lab

**Abbreviations**:

MSc: Master of Science

MS: Master of Science

MPH: Master of Public health

PhD: Doctor of Philosophy

MMED: Master of Medicine

CFAR: Centre for AIDS Research

NTRL: National TB Reference Laboratory

NTLP: National TB and Leprosy Control Program

JCRC: Joint Clinical Research Centre

The collaboration has conducted more than 70 large tuberculosis, HIV and joint HIV /Tuberculosis(TB) studies in the last 35 years and has published more than 360 manuscripts, with its scientists having made numerous contributions as advisors, reviewers, and committee and protocol team members. Important contributions include trials done early in the HIV epidemic confirming severe cutaneous side effects of thiacetazone-containing TB treatment regimens in HIV-infected patients1, the inferiority of ethambutol-based continuation regimens for TB treatment in HIV-infected persons^2^, the critical importance of fully-rifampin based regimens for treating HIV+TB, the safety and efficacy of 3 regimens for treatment of latent TB infection(LTBI) in HIV-infected adults^4^, the first phase 3 treatment stratification trial of shortening TB treatment from 6 to 4 months in HIV-patients with non-cavitary TB who converted their sputum cultures to negative after 2 months^3^, recent TB Trials Consortium(TBTC) studies of the roles of moxifloxacin and higher doses of rifapentine in TB treatment leading up to Study 31^5^,^6^,^7^,^8^,^9^ innovative studies of TB transmission in the community and numerous studies of diagnostics for TB and LTBI, resistance phenotypes against TB infection, and biomarkers of response to TB treatment. Uganda (Makerere University)-Case Western Reserve University Research Collaboration investigators have also made many contributions to clinical trial design and conduct including the importance of detailed Pharmacoki-netics(PK) and Pharmacodynamics (PD) in Early Bactericidal(EBA) studies, novel biomarkers such as sputum Mycobacteria Tuberculosis Messenger Ribonucleic Acid(MTB mRNA), drug packing methods and accountability records, and innovative approaches to community-based Directly Observed Therapy(DOT) during TB treatment trials.

Other areas where the Uganda (Makerere University)-Case Western Reserve University Research Collaboration has been key included matched Uganda-USA fellows research training, as well as clinical disciplines including interventional cardiology, improved management of stroke and other neurological conditions as well as cancer management. In 2015, Drs Emmy Okello and James Kayima (of the Uganda Heart Institute) were trained as interventional cardiologists by Uganda-CWRU collaboration.

Uganda (Makerere University)-Case Western Reserve University Research Collaboration and training has had significant impact on the management of TB/HIV in adults and children globally, such as preventive treatment of tuberculosis (table 2)^4^. Other areas have been the impact on heart disease management in Uganda. The Uganda (Makerere University)-Case Western Reserve University Research Collaboration recently (2020) concluded TBTC study 31 which successfully tested and identified a 4 month Rifapentine Moxifloxacin containing regimen for TB treatment shortening in adults and adolescents with drug sensitive TB^9^. The World health Organization has endorsed this new regimen for the treatment shortening of drug sensitive TB.

**Table 2:** Policy changing key trials conducted by Uganda (Makerere University)-Case Western Reserve University Research Collaboration

Area of Study	Summary results	Impact
**Effect of tuberculosis preventive** **therapy on HIV disease progression and** **survival in HIV-infected adults**. Lim HJ et al, HIV Clin Trials. 2006 Jul- Aug;7(4):172–83.	A study that showed role of MTB preventive treatment on improved outcome in HIV infected persons	Led to early inclusion of INH preventive therapy in the treatment regimen for persons with HIV infection - now incorporated in the national and international Tb prevention regimens in HIV
**Shortening treatment in adults** **with noncavitary tuberculosis and 2-** **month culture conversion**. Johnson JL et alAm J Respir Crit Care Med. 2009 Sep 15;180(6):558–63.	Initial study that showed feasibility of shortening anti TB treatment - that paved way for clinical trials with newer regimens.	Follow up studies have now led to feasibility of a shorter effective 4 month anti Tb regimen
**Four-Month Rifapentine Regimens with** **or without Moxifloxacin for** **Tuberculosis**. Dorman et al. N Engl J Med. 2021 May 6;384(18):1705–18	4 months of daily Rifapentine Moxifloxacin was non-inferior to the 6 months ERHZ regimen for TB treatment in adults and adolescents.	World health Organization has endorsed this new regimen for the treatment shortening of drug sensitive TB.
**Duration of efficacy of treatment of** **latent tuberculosis infection in HIV-** **infected adults**. Johnson JL, Okwera A, Hom DL, Mayanja H, Mutuluuza Kityo C, Nsubuga P, et al. AIDS. 2001 Nov 9;15(16):2137–47	3months of daily Rifampin Isoniazid (3HR) combination conferred long term protection from TB infection compared to 6 months of Isoniazid alone in HIV-infected adults.	3HR regimen was taken up by the NTLP for LTBI treatment.

**Abbreviations**:

HIV: Human Immune Deficiency Virus

MTB: Mycobacteria Tuberculosis

LTBI: latent TB infection

ERHZ: Ethambutol, Rifampin, Isoniazid, Pyrazinamide

HR: Isoniazid, Rifampin

INH: Isoniazid

NTLP: National TB and Leprosy Control Program

JCRC: Joint Clinical Research Centre

## Lessons learned

Over the last 35 years of research, training and mentoring young researchers; the collaboration has the following lessons to share: (i) Training is a key element, as it leads to newer generation of researchers and clinicians to continue with activities. (ii) It is important to conduct research responsive to local health conditions as this has a positive impact on local health outcomes. (iii) Team work is important with equal contributions and benefits to both Ugandan and international collaborators (iv) Mentorship is a key element of all successful research collaborations. (v) High quality research generates knowledge that transforms policy and reflects into a nation's GDP to the benefit of the local communities, (v) Academic institutions present sustainable collaborations for training and research for societal transformation.
